# Lipid Droplets: A New Player in Colorectal Cancer Stem Cells Unveiled by Spectroscopic Imaging

**DOI:** 10.1002/stem.1837

**Published:** 2014-12-18

**Authors:** Luca Tirinato, Carlo Liberale, Simone Di Franco, Patrizio Candeloro, Antonina Benfante, Rosanna La Rocca, Lisette Potze, Roberto Marotta, Roberta Ruffilli, Vijayakumar P Rajamanickam, Mario Malerba, Francesco De Angelis, Andrea Falqui, Ennio Carbone, Matilde Todaro, Jan Paul Medema, Giorgio Stassi, Enzo Di Fabrizio

**Affiliations:** a PSE Division, King Abdullah University of Science and Technology (KAUST)Thuwal, Kingdom of Saudi Arabia; c BESE Division, King Abdullah University of Science and Technology (KAUST)Thuwal, Kingdom of Saudi Arabia; b BioNEM Lab, Department of Experimental and Clinical Medicine, University Magna Graecia of CatanzaroCatanzaro, Italy; d Nanostructures and Istituto Italiano di TecnologiaGenova, Italy; g Nanochemistry, Istituto Italiano di TecnologiaGenova, Italy; e Cellular and Molecular Pathophysiology Laboratory, Department of Surgical and Oncological Sciences, University of PalermoPalermo, Italy; f Laboratory for Experimental Oncology and Radiology (LEXOR), Center for Experimental Molecular Medicine (CEMM), Academic Medical Center (AMC), University of AmsterdamAmsterdam, The Netherlands; h Department of Microbiology Tumor and Cell Biology (MTC), Karolinska InstituteStockholm, Sweden; i Tumor Immunology Lab, Dipartimento di Medicina Sperimentale e Clinica, Università Magna Graecia di CatanzaroCatanzaro, Italy

**Keywords:** Colorectal cancer stem cells, Lipid droplets, Wnt/β-catenin pathway, Raman spectroscopy

## Abstract

The cancer stem cell (CSC) model is describing tumors as a hierarchical organized system and CSCs are suggested to be responsible for cancer recurrence after therapy. The identification of specific markers of CSCs is therefore of paramount importance. Here, we show that high levels of lipid droplets (LDs) are a distinctive mark of CSCs in colorectal (CR) cancer. This increased lipid content was clearly revealed by label-free Raman spectroscopy and it directly correlates with well-accepted CR-CSC markers as CD133 and Wnt pathway activity. By xenotransplantation experiments, we have finally demonstrated that CR-CSCs overexpressing LDs retain most tumorigenic potential. A relevant conceptual advance in this work is the demonstration that a cellular organelle, the LD, is a signature of CSCs, in addition to molecular markers. A further functional characterization of LDs could lead soon to design new target therapies against CR-CSCs. Stem Cells *2015;33:35–44*

## Introduction

Colorectal (CR) cancer is one of the most frequent neoplasms and the second leading cause of cancer-related death in the Western world 1. CR cancer normally originates from clonal expansion of a single intestinal stem or progenitor cell located at the bottom of the CR crypt 2,3 that undergoes genetic and/or epigenetic alterations 4,5. Nevertheless, the hierarchical organization that is present in the crypts as well as the morphogenic signals that sustain this hierarchy appears to be maintained throughout tumor progression 6,7. In agreement, tumors have been shown to contain a hierarchy with a cancer stem cell (CSC) compartment at the apex 8. Importantly, different studies have indicated that these CSCs are more resistant to therapy than differentiated tumor cells 9,10. For these reasons, CR-CSCs have been recognized as key components in CR carcinogenesis and recurrences 11–13. This is why their identification and isolation becomes a crucial step to better understand the mechanisms that underlie their biological behavior 14. To address this issue, one or more of the following in vitro analyses have been used so far: detection of CR-CSC markers, serial colony forming assays, and the propagation as tumor spheres in stem cell culturing conditions 15,16. To prove the tumorigenic potential of the isolated CR-CSCs, it is then necessary to perform serial injections of the spheres into immune compromised mice 17. Unfortunately, nearly all the potential markers of CR-CSCs so far proposed, such as CD133 17,18, CD44 19, ESA (EpCAM) 18, CD166 19, ALDH-1^9^, Musashi 1 (Msi1) 20, and LGR5 21 require staining and are not completely unique for the CSC population.

It is therefore highly desirable to develop an alternative, rapid, and reliable technique for CR-CSC detection and sorting. The identification of such a method could also reveal new relevant cellular/functional aspects of the CSC subpopulation 14. Raman techniques have been recently used for biological and medical studies as they display, due to the sensitivity to the chemical structure of biomolecules, non-perturbative sampling capabilities, label-free imaging, and high spatial resolution 22–24. For instance, Raman microspectroscopy has been used to study DNA and protein distribution inside cells 25,26, the cellular uptake and distribution of liposomal drug carriers 27, label-free mitochondrial distribution 28, lipidomic in leukocytes 29, and lipid imaging in human lung-cancer cells and in brain tissues 30.

In this study, Raman spectroscopy, fluorescence microscopy, flow-cytometry, and electron microscopy are used to investigate the presence of distinctive features of CR-CSCs compared to differentiated tumor cells and normal epithelial colon cells. We show that Raman microspectroscopy highlights a higher content of lipids in CR-CSCs compared to the differentiated counterpart and normal CR cells. Fluorescence microscopy with hydrophobic dyes, BODIPY 31, and LD540 32 clearly identifies the origin of the larger lipid content as an increased expression of lipid droplets (LDs). The large amount of LDs is also confirmed and quantified by flow cytometry and electron microscopy. As a remarkable point, we find that LD content in CSC subpopulation is directly correlated with the over-expression of CD133 and high Wnt/β-catenin pathway activity, two well-accepted markers for CR-CSCs. The correlation between LD content and tumorigenic potential was checked through the injection of different subsets (LDs^High^/LDs^Low^) in NOD/SCID mice.

From a detection point of view, the large amount of LDs produces remarkable increased intensities of the Raman peaks corresponding to specific vibrations of fatty acids, and the intensity differences are so unambiguously evident that these Raman modes are ideal candidates as Raman markers for a fast, robust, and label-free method for CR-CSC identification. From a biological/functional point of view, LDs can be an ideal target for future colon cancer therapies.

## Materials and Methods

### Cell Cultures

CR-CSC cultures were generated as previously described by Ricci-Vitiani *et al.* 6,18 and cultured in ultra-low adhesion flasks (Corning, Lowell, MA, http://www.corning.com) in Dulbecco's modified Eagle's medium (DMEM)/F-12 serum-free medium (Life Technologies, Carlsbad, CA, http://www.lifetechnologies.com) supplemented with fresh epidermal growth factor (EGF) (20 ng/ml) and basic fibroblast growth factor (FGF) (10 ng/ml) (Sigma-Aldrich, St. Louis, MO, http://www.sigmaaldrich.com) to promote their growth. A GFP^+^ subculture was obtained by lentiviral transduction as previously described 6. Differentiated cells (sphere-derived adherent cells [SDACs]) were obtained by dissociating CR-CSCs and culturing them in Dulbecco's modified medium supplemented with 10% fetal calf serum (FCS) in adherent conditions for at least 25 days. Normal epithelial colon cells (NECCs) (CCD841-CoN, Manassas, VA, www.atcc.org) and colon carcinoma cell (CCC) lines (HCT116 and RKO, Manassas, VA, www.atcc.org) were cultured in RPMI and α-MEM completed with 10% of fetal bovine serum (FBS) and 1% of P/S, respectively.

### Raman Measurements

Raman microspectroscopy is carried out by means of a Renishaw InVia Raman microscope (Wotton Under Edge, UK, www.renishaw.com), equipped with a motorized stage for the laser-scanning of the sample. The excitation wavelength is 633 nm and the incident light is focused on the sample through an Olympus (Olympus Italia S.r.l., Segrate, Italy, www.olympus-europa.com) ×60/1.0 NA water immersion objective. The laser power at the sample level is about 3 mW. A notch-filter is used to block the Rayleigh back-scattered light. Before recording Raman measurements, the cells are passaged by trypsinization, washed three times with phosphate buffered saline (PBS), and then resuspended in the same buffer. During the measurements, CaF_2_ slides are used as substrates because of their negligible Raman signal background.

For the imaging experiments, cells were scanned through the laser focus in a raster pattern with a typical step-size of 1 µm. Raman spectra are recorded in the 800–3,200 cm^−1^ range and the accumulation time is 5 seconds per each pixel. Subsequently, Raman images were created by plotting the integrated intensity of a specific Raman band as a function of position. Since different biomolecules exhibit different characteristic Raman bands, this technique allows for a label-free imaging of the spatial distribution of biomolecules inside the cell.

### Confocal Microscopy

Fluorescence images have been collected using a Nikon A1 confocal-laser-scanning microscope (Tokyo, Japan, www.nikon.com) with a PlanApo ×60 oil immersion objective with a 1.40 numerical aperture. In suspension live cells have been stained for LDs using BODIPY 493/503 (Molecular Probes, Invitrogen, Carlsbad, CA, http://www.lifetechnologies.com). BODIPY 493/503 was used at 1 µg/ml. Cells were washed with PBS 1× and incubated with BODIPY 493/503 for 15 minutes at room temperature.

### Flow Cytometry

All cells were collected from the flasks, washed with PBS 1×, and incubated with BODIPY 493/503 at 1 µg/ml or LD540 at 0.1 µg/ml for 15 and 10 minutes, respectively, at room temperature in the dark. CD133 was stained using an anti-CD133 antibody (MiltenyiBiotec, Bergisch Gladbach, Germany, http://www.miltenyibiotec.com) allophycocyanin (APC)-conjugated (Invitrogen, Carlsbad, CA, http://www.lifetechnologies.com/nl/en/home/brands/invitrogen.html). Stained cells were washed twice with PBS 1× and resuspended in the same solution. Samples were analyzed by FACSAria II flow cytometer (BD Biosciences, San Jose, CA, www.bd.com). To allow for comparison of the different cell lines, gains for forward-scattering, side-scattering, and fluorescence photo multiplier tubes are kept the same on all the measurements.

### Transmission Electron Microscopy Measurements

NECCs, CR-CSCs, SDACs, and CCCs were processed for transmission electron microscopy. The volume fraction of the cell occupied by LDs was estimated using point counting stereology techniques (for more details see Supporting Information Methods).

### Cell Sorting

Two different CR-CSC lines bearing the TCF Optimal Promoter (TOP)-green fluorescent protein (GFP) construct were collected from the flasks and sorted for GFP^High^ and GFP^Low^ (both sorted fractions consist of approximately 11%–13% of the total GFP^+^ population), using a fluorescence-activated cell sorting (FACS) FACSAria II, and then stained for LD content using the LD540 dye.

### Limiting Dilution Assay

The self-renewal capacity of the CR-CSC LDs^High^ and LDs^Low^ was assayed by dissociation of colon cancer spheroids and plating cells at serial dilution (1, 2, 4, 8, 16, 32, 64, and 128 cells per well) in 96-well microplate with flat bottom and repellent surface for low attachment (CELLSTAR Cell-Repellent Surface, Greiner Bio-One, UK, http://www.selectscience.net/products/cellstar-cell-repellent-surface/?prodID=171921). The cell culture medium used for this assay is the CSC medium described above (DMEM/F-12 serum-free medium, supplemented with fresh EGF and basic FGF). Results were statistically evaluated after 4 weeks using the Extreme Limiting Dilution Analysis (ELDA) software 33.

### In Vivo Tumorigenicity Assay

Mice experiments were performed according to the animal care committee guidelines of the University of Palermo. For in vivo limiting-dilution injection, total CR-CSC population was sorted for LD540 intensity and 100, 500, 1,000, and 8,000 cells from 12% lowest and 12% highest were deposited by FACS in a 96-well plate containing stem cell medium, admixed with matrigel, and then injected subcutaneously in 5-week-old NOD/SCID mice (Charles River Laboratories, IT, http://www.criver.com). Tumor size was measured weekly using an electronic caliper and the volume was then calculated with the formula: larger diameter × (smaller diameter)^2^ × *π*/6. At the end of the experiments, mice were sacrificed and tumors collected. Tumor tissues were finally processed for morphological and immune-histochemical analysis or for in vitro culture.

### Statistical Analysis

Most of the Raman spectra presented in this work are average curves coming from a large number of measurements. Standard deviation and principal component analysis (for more details see Supporting Information) were used for Raman analysis.

Transmission electron microscopy (TEM) comparison of the volume fraction of cell occupied by LDs for each sample was done with Student's *t* test (for more details see Supporting Information). For clonogenic assay, the statistical analysis was performed with Prism 5 (GraphPad Software, La Jolla, CA, http://www.graphpad.com) applying Bonferroni Multiple Comparison Test. Differences were considered significant with *p*-values <.05 (*) and <.01 (**).

## Results

### CR-CSCs Show a Specific Lipid Raman Signature

**Figure 1:**
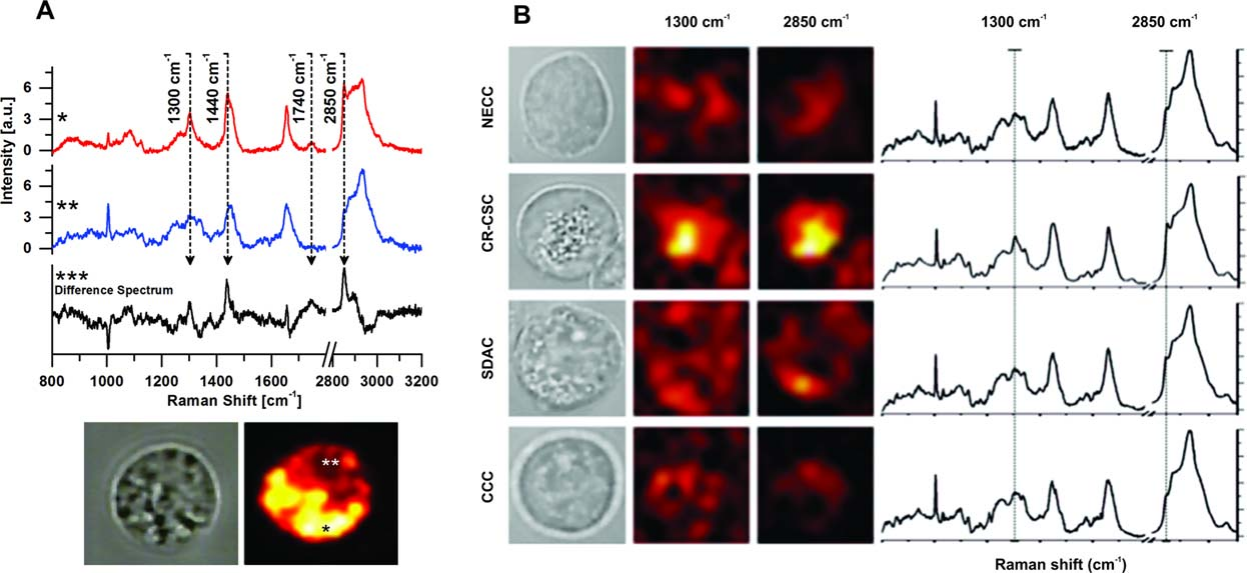
Raman characterization and mapping of colon cell samples. (A): Two different cell regions, indicated as * and **, respectively, can be clearly identified in the cell according to their Raman spectra. The Raman differences are due to four main peaks located at 1,300, 1,440, 1,740, and 2,850 cm^−1^. Typical spectra from region * (top curve) and region ** (bottom curve) show that region * has higher expression of all these aforementioned peaks compared to region **, as clearly highlighted by the Raman difference spectrum ***; brightfield image of a CR-CSC and Raman imaging at 2,850 cm^−1^ of the same cell are reported, highlighting the two different regions. (B): From the top row to the bottom one: NECCs, CR-CSCs, SDACs, and CCCs. Brightfield images are reported on the first column, while Raman images calculated at 1,300 and 2,850 cm^−1^ are reported in the second and third column, respectively. The fourth column shows spectra averaged over the whole cell area, for each cell line. Raman images in the second column are similar to the corresponding ones of the third column, thus revealing that the two Raman modes at 1,300 and 2,850 cm^−1^ are space-correlated. Peaks related to lipidic vibrations are more pronounced in the CR-CSCs (second row) compared to SDACs and CCCs, as it is evident both from Raman images and spectra. NECCs reported in the first row express the lowest Raman intensities of lipidic vibrations. Abbreviations: CCC, colon carcinoma cell; CR-CSC, colorectal cancer stem cell; NECC, normal epithelial colon cell; SDAC, sphere-derived adherent cell.

Primary CR-CSC lines, characterized for CD133 18 expression and high Wnt/β-catenin pathway activity 6, and SDACs from distinct human CR cancer specimens derived from seven patients (stage II-IV) undergoing CR resection (see Supporting Information) were analyzed by Raman spectroscopy. In addition, NECCs and two CCCs were used for comparison. Figure 1A shows a typical Raman imaging result recorded on a single CR-CSC. When analyzing the spectra measured across the cell area, two spatial regions (named region * and **) with different Raman features were identified. The characteristic Raman spectra (Fig. 1A*, 1A**) from these regions exhibit clear differences for peak intensity at 1,300, 1,440, and 1,740 cm^−1^ and for the Raman band at 2,800–3,000 cm^−1^. The assignment of all these Raman bands has been thoroughly discussed in literature, with the peaks at 1,300 and 1,740 cm^−1^ unambiguously assigned to molecular vibrations of lipids 34,35, while the 1,440–1,450 cm^−1^ and 2,800–3,000 cm^−1^ bands are indicators for the lipid to protein ratio (see Supporting Information).

Comparison of the two spectra (Fig. 1A***) revealed that region * has a larger content of lipids. Besides the intensity increase observed for the peaks at 1,300 and 1,740 cm^−1^, which are characteristic only of lipids, both the aforementioned lipid to protein indicators (1,440–1,450 and 2,800–3,000 cm^−1^ bands) denote larger lipid content. In fact, a detailed analysis of the 1,440–1,450 cm^−1^ band shows a shift toward the 1,440 cm^−1^ vibration typical of lipids, while in the 2,800–3,000 cm^−1^ region the CH_2_ symmetric stretching at 2,850 cm^−1^ is noticeably more pronounced (CH_2_ groups are more frequent in fatty acids than in proteins). Overlapping the bright-field image of the cell with the Raman map at 2,850 cm^−1^ clearly shows that these lipid-rich areas correspond to the presence of granulated (or droplet-like) morphological structures. Also, imaging at 1,300, 1,440, and 1,740 cm^−1^ (data not shown) exhibits the same spatial correlation with the bright-field image. These peaks are therefore spatially overlapping, which confirmed the lipid nature of the observed droplets.

Figure 1B shows the typical Raman imaging for all the measured cell lines, with intensity maps at 1,300 and 2,850 cm^−1^, along with whole-cell-averaged Raman spectra on the last column. CR-CSCs clearly exhibit a distinctive Raman signature with remarkable intensities for the two aforementioned peaks. Again, these features are localized in spatial regions corresponding to granules observed in the bright-field image of the cell. The SDACs have partially inherited this characteristic, but at a smaller extent. Even if some spots are still noticeable in the bright-field picture of the cell (mostly in the left-bottom part of the SDACs in Fig. 1B), the peaks intensities at 1,300 and 2,850 cm^−1^, on the averaged Raman spectra, are much smaller compared to CR-CSCs. CCCs exhibit, instead, few spots. Accordingly, Raman intensities at 1,300 and 2,850 cm^−1^ drop to smaller values, and Raman spectra from CCCs generally resemble to Raman profile of region ** of Figure 1A. Finally, the NECCs (first row in Fig. 1B) show the most uniform appearance, with a nearly absence of spots in the bright-field image, and also their Raman spectra have small intensities at the characteristic frequencies of lipids vibrations.

In order to prove that Raman spectroscopy can provide a fast tool for CR-CSC detection (and for future sorting applications) we extended our measurements. Besides point-by-point Raman mappings, we measured a single Raman spectrum in the 800–1,800 cm^−1^ range for each cell using a line-focused laser excitation extending for the whole cell diameter. In these measurements (see also Supporting Information Fig. S1, S2), the 1,300 cm^−1^ Raman peak of CR-CSCs displayed an intensity level that was much more pronounced than in normal cells or in other non-stem CR cancer cell lines, suggesting that it can be used as a Raman marker for detecting CR-CSCs. We noticed that, due to the clear change in spectra between CR-CSCs and the other cell types, no data treatments were necessary (we reported Principal Component Analysis in Supporting Information Fig. S3 just to point out the sensitivity of the method 36).

### LD Quantification

To confirm the presence, and to assess the amount of the lipid-rich regions revealed by Raman spectroscopy, fluorescence imaging and flow cytometer measurements were performed on the cell samples using BODIPY 493/503 or LD540 staining, which are consolidated dyes for cellular LD visualization 32,37,38.

**Figure 2:**
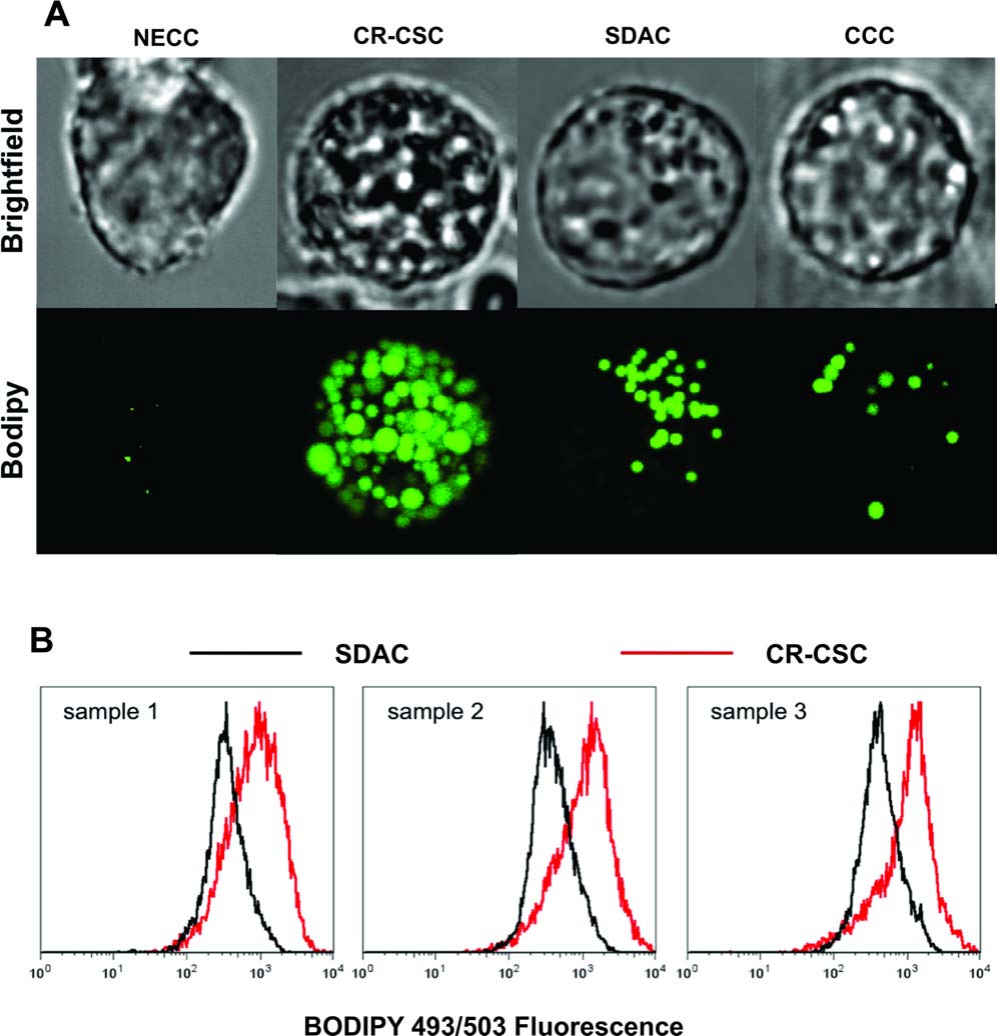
Lipid droplet quantification. (A): Comparison of typical z-projected confocal fluorescence images of the investigated cell lines stained with BODIPY 493/503. The lipid droplet content on CR-CSCs is higher compared to all the other cell lines. (B): Histograms overlay for flow-cytometry BODIPY fluorescence measurements regarding three of the CR-CSC (red) lines and their SDACs (black). Abbreviations: CCC, colon carcinoma cell; CR-CSC, colorectal cancer stem cell; NECC, normal epithelial colon cell; SDAC, sphere-derived adherent cell.

Confocal images were collected for all the colon cell lines and z-projections created using the ImageJ software 39. The acquired images clearly showed the “lipid droplet” nature of the same granular structures visible in the bright-field image, which are responsible for the high lipid-related Raman peaks. A comparison of typical LD content among the considered samples is shown in Figure 2A and Supporting Information Figure S4. From this it is clear that the number of LDs increases from the normal cells to the CR-CSCs.

Moreover, flow-cytometric analysis allows for a statistical assessment of the LD expression difference among the investigated cell lines. Comparison of histograms for CR-CSC lines from three different patients and their related SDACs confirmed the higher LD expression in multiple CR-CSCs.

**Figure 3:**
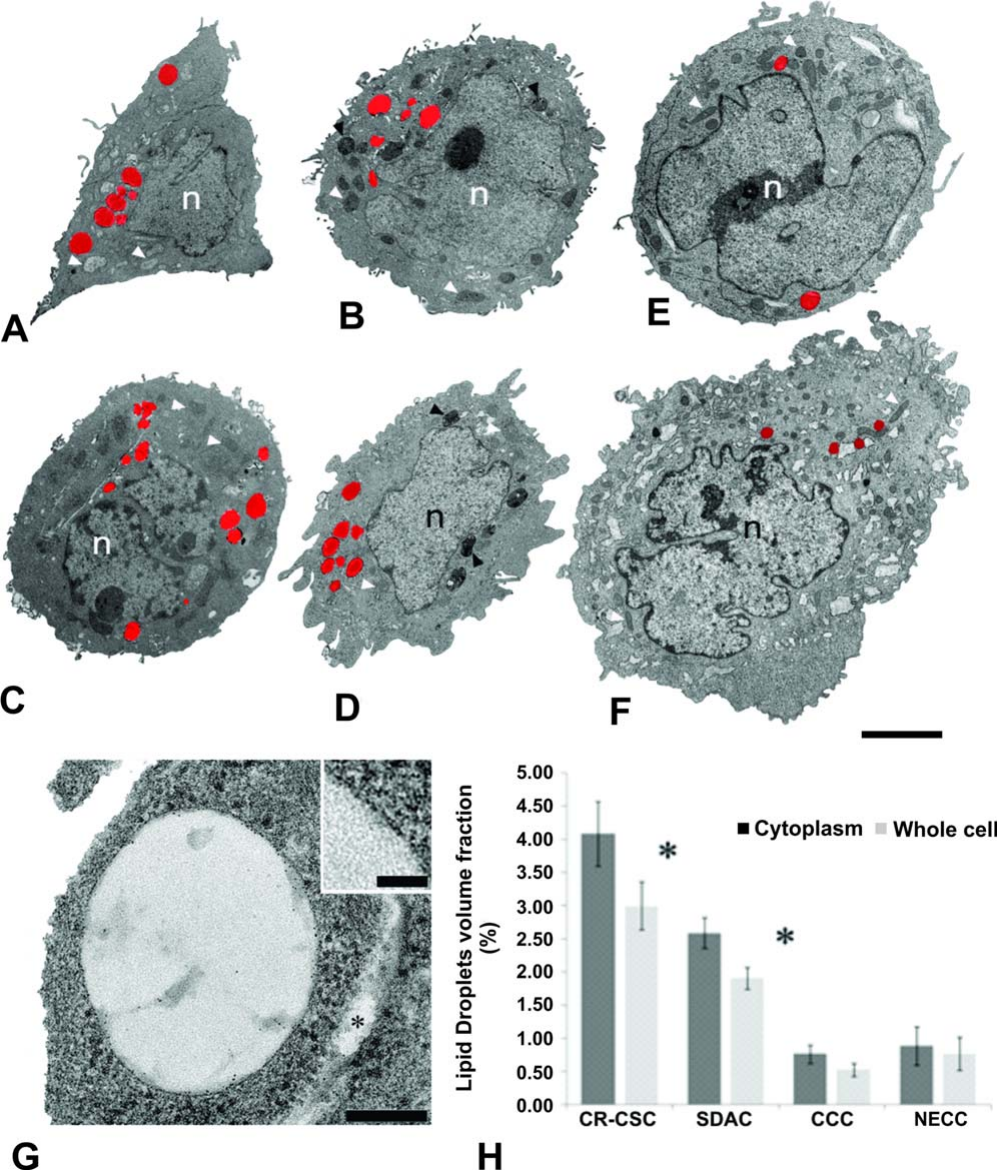
Transmission electron microscopy (TEM) to reveal lipid droplets on colorectal cells. (A–F): TEM images of the CR-CSCs, their differentiated forms (SDACs), CCCs, and NECCs. (A and B), parasagittal sections of (A) CR-CSCs and (B) SDACs belonging to human patient 1; (C and D), parasagittal sections of (C) CR-CSCs and (D) SDACs belonging to human patient 2; (E), parasagittal section of a CCC; (F), parasagittal section of a NECC. White arrowheads point to mitochondria; black arrowheads point to multivesicular bodies and late-endosome/lysosome hybrids. LDs are colored in red. n, nucleus. (G): TEM image of a LD in cross-section. The asterisk points to the endoplasmic reticulum. Inset: detail of the LD single membrane leaflet. (H): Volume fraction of LDs in the cytoplasm and in the whole cell. Error bars, SEM. Statistical significance is denoted by * (*p* ≤ .01, Student's *t* test). Scale bars are 4 µm for A–F and 100 nm for G. Abbreviations: CCC, colon carcinoma cell; CR-CSC, colorectal cancer stem cell; NECC, normal epithelial colon cell; SDAC, sphere-derived adherent cell.

The ultra-structural analysis performed with TEM on NECCs, two different CR-CSC samples, their relative SDACs, and CCCs, corroborated both Raman and fluorescent microscopy results (Fig. 3). The LDs were unambiguously identified in the cell cytoplasm, often close to the endoplasmic reticulum (Supporting Information Fig. S5), as subcellular structures delimited by a single membrane leaflet (Fig. 3G and inset) 37. The stereological analysis, performed to quantify the volume fraction of the LDs in the various cell lines analyzed, further confirmed the insights from Raman and fluorescent microscopy (Fig. 3H). We measured a LD volume fraction expressed as percentage of cytoplasmic volume ranging from 4.09% ± 0.48%, for the CR-CSCs, to 0.89% ± 0.29%, for the NECCs, value that fall into the range reported for other cell types (Fig. 3H) 40. In the CR-CSCs, the LD volume fraction in the whole cell and in the cytoplasm resulted to be significantly higher (*p* ≤ .01), compared to that measured for SDACs (Fig. 3H). Furthermore, the LD volume fraction in the SDACs was largely higher (*p* ≤ .01) compared to that measured inside the NECCs and the CCCs (Fig. 3H).

### Correlation Between CD133, Wnt, and LDs

**Figure 4:**
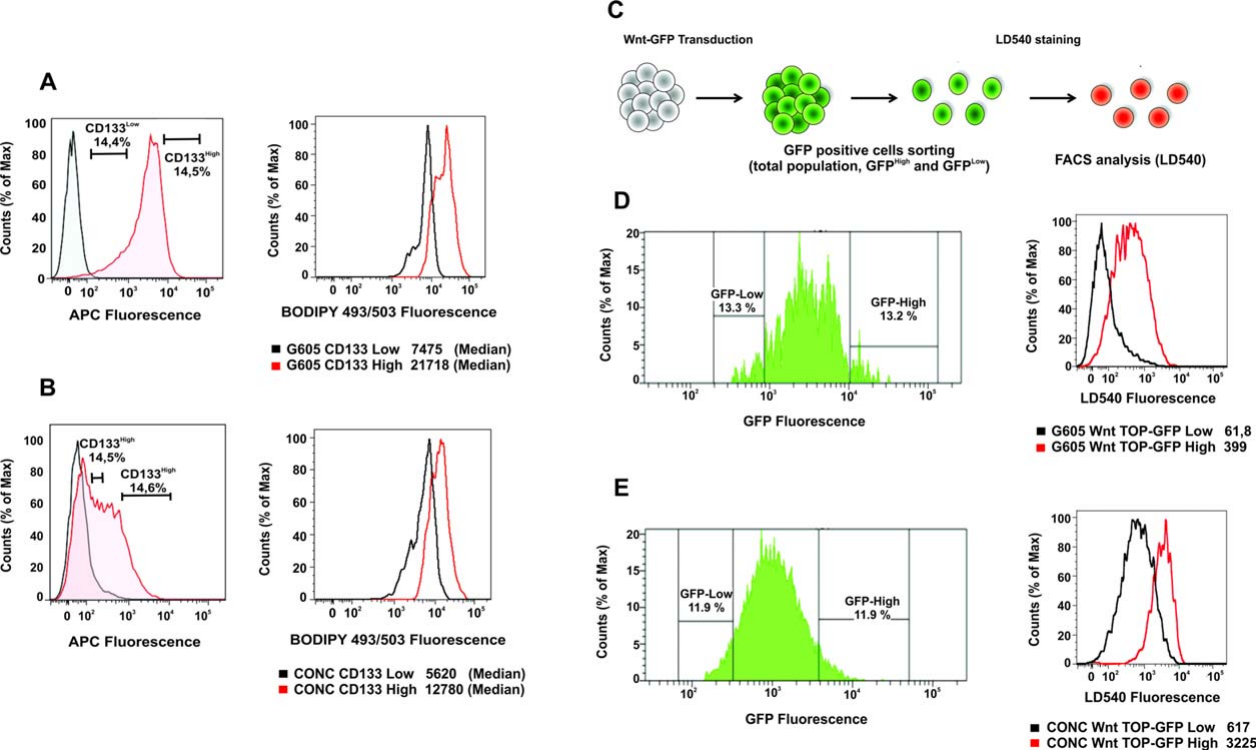
Correlation of the expression levels of the Lipid Droplets with CD133 and Wnt/β-catenin. (A, B): The expression of the LDs in CD133^High^ and CD133^Low^ cells were analyzed by flow cytometry. Cells were stained with an anti-CD133 APC-conjugated and then with BODIPY 493/503. Both CD133^High^ samples (A and B red lines) have a higher expression of LDs compared to the CD133^Low^ (black lines). (C): Schematic representation of the TOP-GFP Wnt construct. (D, E): Cells were sorted for GFP expression (GFP^High^ and GFP^Low^) and then stained for LDs with LD540 dye; both TOP-GFP samples have the same behavior showing as Wnt/β-catenin pathway expression clearly correlates with LDs quantity. Abbreviations: APC, allophycocyanin; FACS, fluorescence-activated cell sorting; GFP, green fluorescent protein; TOP, TCF Optimal Promoter (TOP).

To verify whether LD content and the expression of CR-CSC markers directly correlate, we performed flow cytometer measurements of CD133 expression and Wnt/β-catenin pathway activity. In a first experiment, different CR-CSC samples were double-stained for LDs and CD133 with BODIPY 493/503 and anti-CD133 antibody. Flow cytometric analysis (Fig. 4A, 4B) showed a clear correlation between the two markers. In a second experiment, LDs and Wnt correlation was studied using two CR-CSC cultures with a TOP-GFP reporter gene 6. Importantly, cells derived from these single-cell cloned TOP-GFP cultures still showed a big heterogeneity in Wnt signaling level 6. The two cell lines were sorted based on the GFP fluorescence, as an indicator of Wnt activity, into two subsets, Wnt^High^ and Wnt^Low^. Sorted cells were then stained for LD content using the LD540 dye, taking advantage of the fact that it can be used in combination with GFP (green) since its emission spectrum extends to red fluorescence (Fig. 4C–4E). It is evident that LD expression and Wnt signaling level strongly correlate. It is important to note that the different expression of LDs is not due to the use of different cell media, since Wnt^High^ and Wnt^Low^ cells were sorted from the same population, such as for the case of CD133, as reported above. These results, showing a clear correlation between CD133, Wnt, and LD content, indicate that LDs could be used as CR-CSC marker, and suggest a possible functional or metabolic link of LDs in CR-CSCs 41,42.

### A High LD Content Is Linked to Clonogenic Potential of CR-CSCs

**Figure 5:**
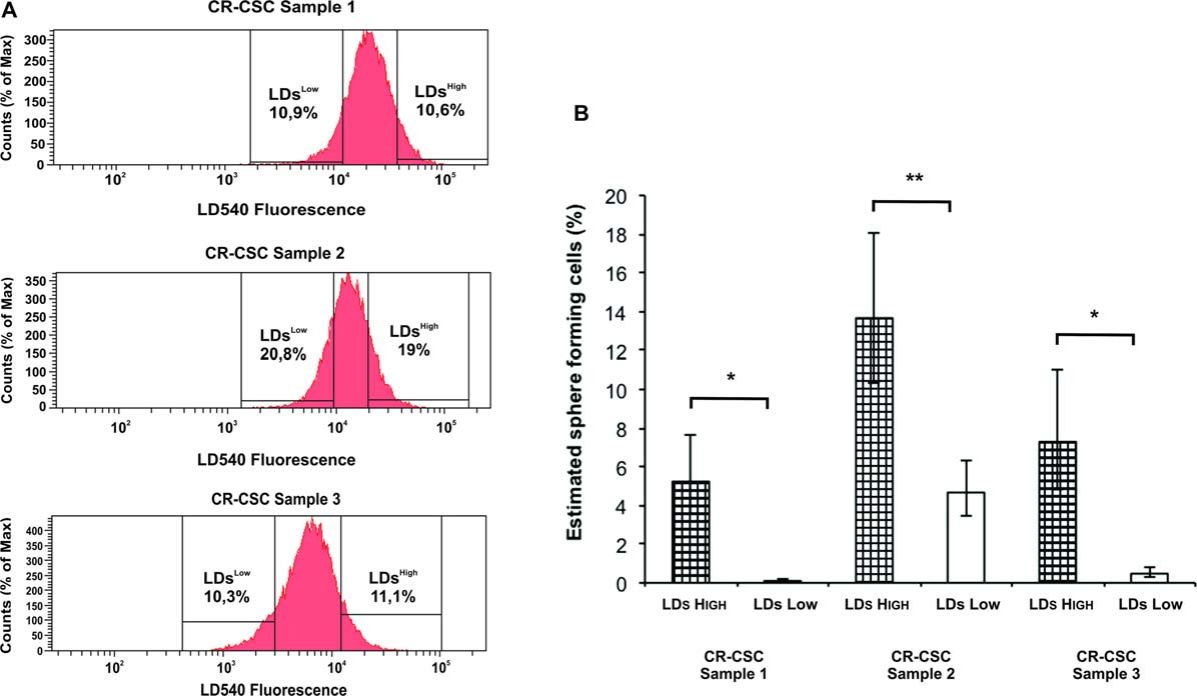
Clonogenic assay of CR-CSC LDs^High/Low^ subpopulations in vitro. Three different CR-CSC samples were tested for their clonogenic potential. (A): CR-CSCs were sorted for LDs^High^ and LDs^Low^ by fluorescence activated cell sorting for LDs using LD540 dye and then deposited 1, 2, 4, 8, 16, 32, 64, and 128 cells per well. (B): The estimated sphere-forming cells were analyzed using the extreme limiting dilution analysis as reported in the graph. All the three LDs^High^ cell samples have a significantly increased clonogenic potential compared to the LDs^Low^ cell samples. Error bars representing the SD of the mean of three independent experiments are shown. Significance is indicated (*, *p* < .05 and **, *p* < .01). Abbreviations: CR-CSC, colorectal cancer stem cell; LD, lipid droplet.

Different CR-CSC lines were stained with the LD540 dye and sorted in LDs^High^ and LDs^Low^ populations. The sorted cells were used to perform a limiting dilution assay (LDA) to test the clonogenic potential. The results reported in Figure 5 show that LDs^High^ cells possess a higher clonogenic potential compared to the LDs^Low^ in all the CR-CSC lines analyzed, indicating that LD content correlates with clonogenicity. In addition, this may suggest a possible role of these LDs in giving an advantage in promoting and sustaining cell growth. These data show that CR cells contain a subpopulation of cells with high levels of LDs that can be used as a marker to single out the CSC subset present within heterogenic tumor cell population.

### In Vivo Tumorigenic Potential Analysis

**Figure 6:**
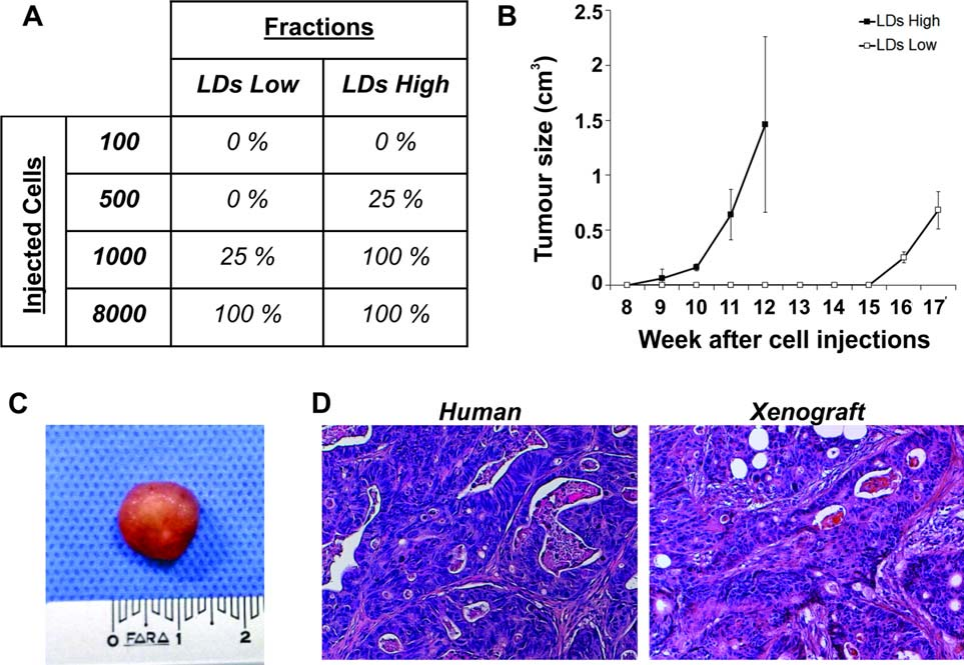
Cells with high lipid droplet content show cancer stem cell (CSC) tumorigenic features in vivo. (A): In vivo transfer of LD540 stained cultures. Different cell numbers from the indicated populations were injected into NOD/SCID mice after fluorescence-activated cell sorting. The percentage of successful tumor initiations after 17 weeks out of four injections for each condition is shown. The LDs^High^ fraction showed the highest tumor-initiating capacity (refer Fig. 6B for the kinetic of tumor appearance). (B): Time course of colon tumor growth after injection of 8,000 LDs^High^ or 8,000 LDs^Low^ CR-CSCs (tumor volumes were determined as described in Materials and Methods). (C): Representative picture of the xenotransplanted tumors. (D): Hematoxylin/eosin staining of xenografts shows clear evidence of colon carcinoma morphology, resembling the human parental phenotype. Abbreviation: LD, lipid droplet.

To determine whether CR-CSC LDs^High^ and LDs^Low^ fractions display tumorigenic potential, we injected CR cancer cells subcutaneously in immune-compromised mice. The experiment was performed using four different cellular dilutions (8,000, 1,000, 500, and 100 cells), for both LDs^High^ and LDs^Low^ fractions, and following the tumor growth over time after cells injection. Although both CR-CSC LDs^High^ and LDs^Low^ exhibited tumorigenic activity, LDs^Low^ cells generated delayed small tumors, whereas the LDs^High^ cell fraction was able to grow as large tumors (Fig. 6A, 6B). Such a low tumorigenic activity of the LDs^Low^ population was lost when a small number of cells (≤500 cells) were subcutaneously injected, suggesting that cells endowed with tumorigenic potential are included into the LDs^High^ population, while LDs^Low^ cells could represent the more differentiated nontumorigenic cell population (Fig. 6A). Of note, 17 weeks later, we observed that xenograft tumors recapitulated the morphological features of the parental CR tumors (Fig. 6C, 6D).

## Discussion

In the last years, it was shown that CR cancer cells exhibit more LDs than their normal counterpart 41,43, but here we have found that CR-CSCs can be identified for having the largest amount of LDs when compared with differentiated tumor or normal epithelial cells. This finding has been confirmed by measurements carried out on CR-CSCs from seven different patients and a well-studied TOP-GFP reported gene cell system for Wnt/β-catenin pathway activity 6. We have demonstrated through an in vivo assay that, even though some CR-CSC LDs^Low^ could be tumorigenic and develop slowly growing small tumors, most of the tumorigenic potential is restricted to the CR-CSC LDs^High^ fraction.

We can speculate that the higher expression of LDs in CR-CSCs could be part of the disease pathogenesis confirming the increasing interest toward these organelles, shown by the recent literature 41,42. Even if LD role is not yet clear, there are evidences of the over-expression of these organelles in colon cancer development. LD accumulation was indeed found in polyp epithelium of Apc^Min^ mice, suggesting that they may contribute to polyp development 44.

It is known that LDs in neoplastic cells act as distinct intracellular domain for regulated eicosanoids production (Prostaglandin E2) starting from arachidonic acid (AA) 43. The metabolism of AA is directly implicated in the generation of a chronic inflammatory tissue environment that could promote carcinogenesis. For instance, 80%–90% of colon carcinomas show an enhanced cyclooxygenase-2 (COX-2; prostaglandin H synthase) expression compared with normal intestinal mucosa 45–47. COX-2 is the enzyme that catalyzes the rate-limiting step in eicosanoids synthesis, converting AA into prostaglandins. Our results could therefore point to a potential link between LDs over-expressing CR-CSCs and inflammation in cancer.

Additionally, besides their function in the generation of eicosanoids, LDs constitute sites of compartmentalization of several signaling-relevant proteins, which may have functions beyond AA metabolism. Indeed, proteins with well-established roles in oncogenic cell transformation, tumorigenesis, and metastasis, or identified as potential CR cancer biomarkers including PI3K, ERK2, p38, PKC, caveolin, and ADRP, were shown to localize in LDs in a variety of cell types 48–51.

Although no specific LD inhibitors have been described so far, different classes of drugs have been demonstrated to inhibit diverse lipid pathways, both in a direct or indirect way; some of them, such as nonsteroidal anti-inflammatory drugs 52,53 and statins 54 could interfere with LD formation in vitro and in vivo. Even if the mechanism of action of these drugs is not completely understood, they have exhibited successful results in the prevention of the CR cancer, suggesting a pivotal role for the LDs in CR-CSCs. Moreover, very recent results are indicating an even tighter connection between lipid metabolism and stemness 55,56.

The biological implications of LD overexpression in CR-CSCs need further investigations. On this side, we could just speculate that the higher content of LDs may be crucial for CR-CSCs in giving them an advantage in proliferation 57, as an energy reserve to resist starvation, to survive in prohibitive microenvironment conditions (oxidative/energy stress), or even stimulating signaling pathways promoting cell invasion 58.

In this work, we also propose a new method, Raman spectroscopy, to highlight and characterize CR cancer stemness. While, at the moment, there are no procedures available for clinical use based on this label-free technique, a quick look to the recent scientific literature shows that pivotal achievements on the technical side have been accomplished 59,60 and we believe that a clear track has been traced to make it an effective tool in clinical diagnosis. Differently from label-based fluorescence analysis, the Raman technique collects the whole spectral content without any additional tagging procedure, and without any external perturbation of the cell biological machinery, becoming ideal for in vivo multimolecular detection. It is worth noticing that several research groups are working to develop high sensitivity Raman-based microendoscopes for future in vivo screening 59,60. These technical achievements, combined with our finding about the abundance of LDs (that are an ideal target for Raman detection) in CR-CSCs, place a solid ground to our view that a Raman clinical application is reasonably within the reach.

## Conclusions

Our data put in evidence that LDs could be considered as new and important “players” in CR-CSCs, and a rather interesting cellular target for future innovative anticancer therapies. Moreover, we propose identification of CR-CSCs through Raman spectroscopy, a label-free technique, able to visualize the LD cell content.
