# Supplementary figures and images for: Lipid Droplets: A New Player in Colorectal Cancer Stem Cells Unveiled by Spectroscopic Imaging
Source: Stem Cells. 2014 Dec 18;33(1):35–44. doi: 10.1002/stem.1837 (PMC4311668; doi:10.1002/stem.1837)

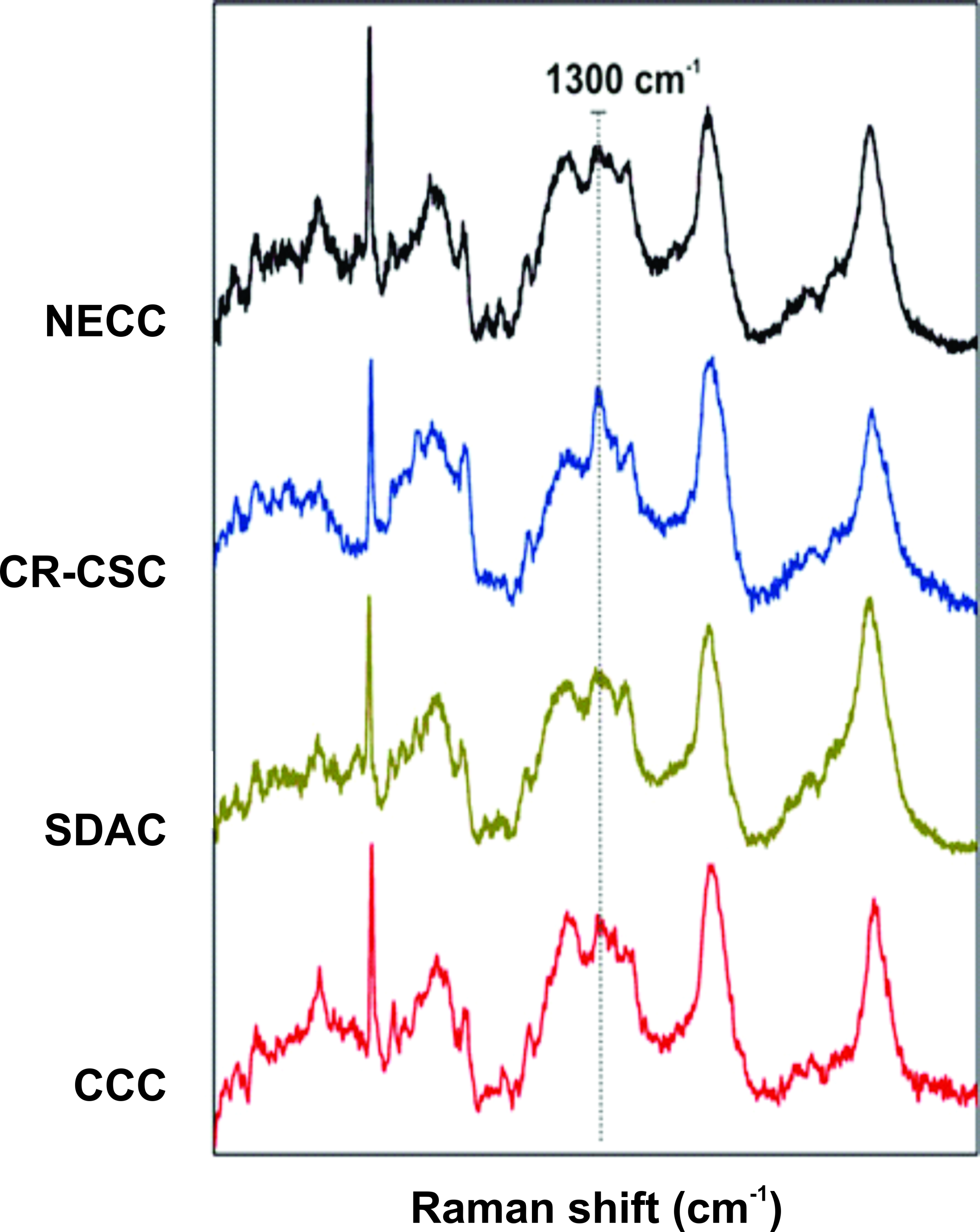

Supplement: Supplementary file 1 — Supporting Figure 1 [file stem0033-0035-sd1.tif]

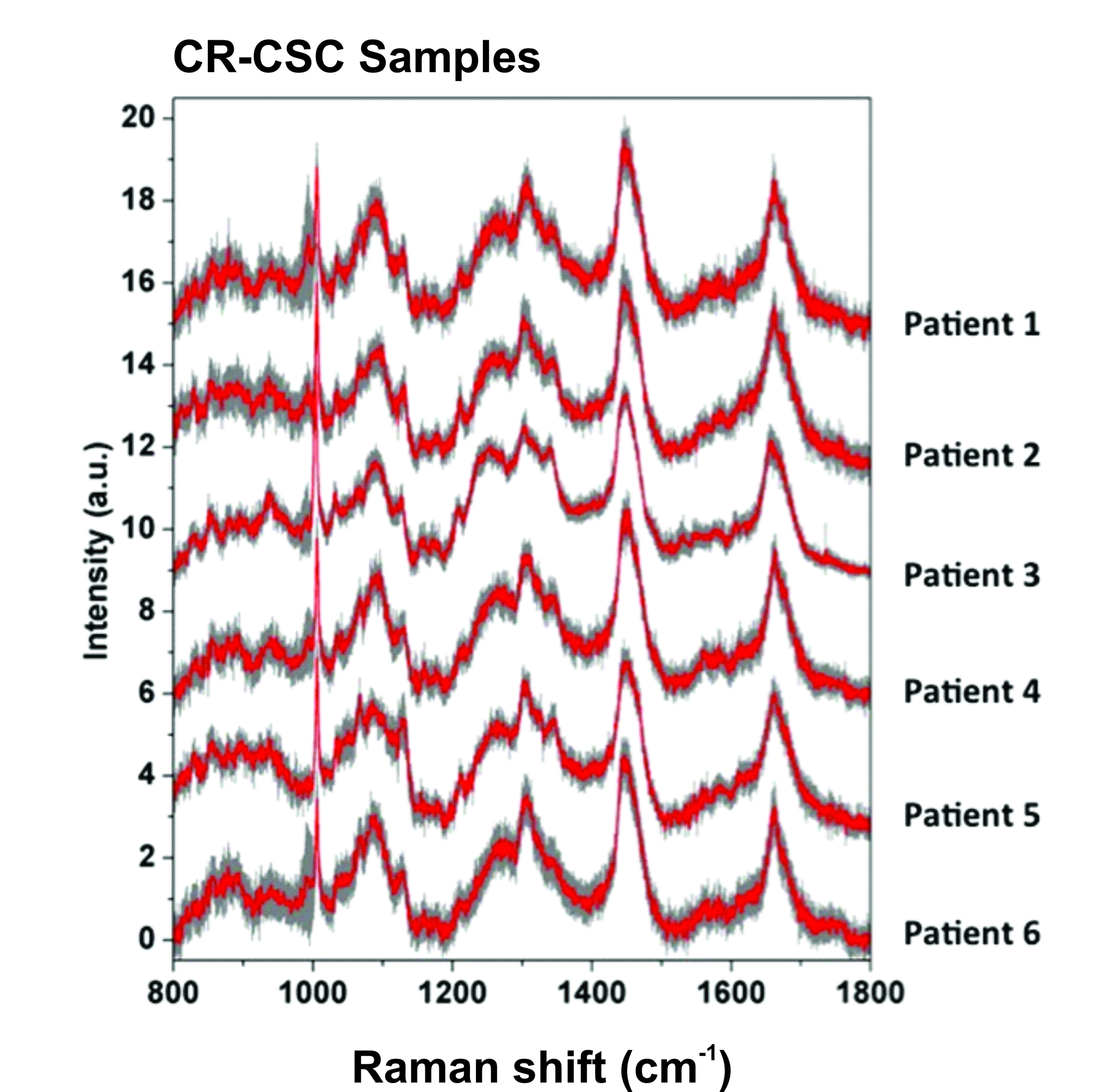

Supplement: Supplementary file 2 — Supporting Figure 2 [file stem0033-0035-sd2.tif]

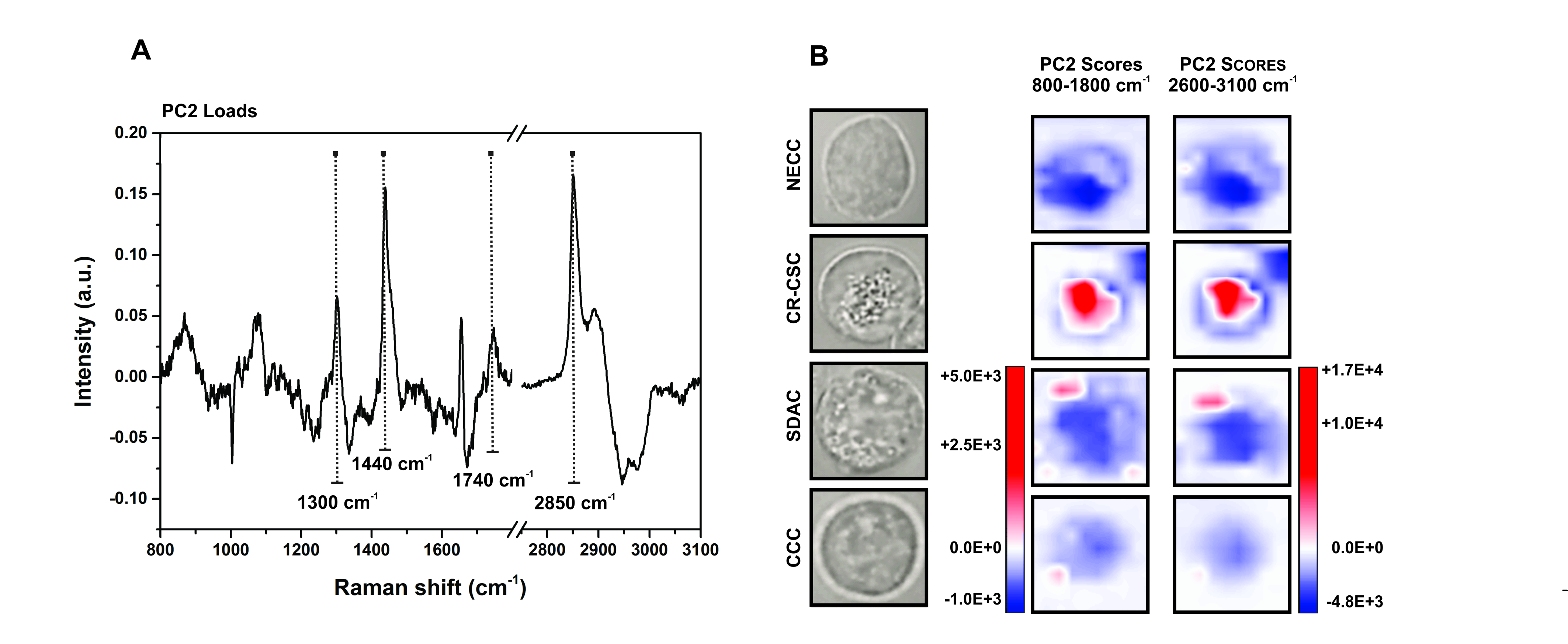

Supplement: Supplementary file 3 — Supporting Figure 3 [file stem0033-0035-sd3.tif]

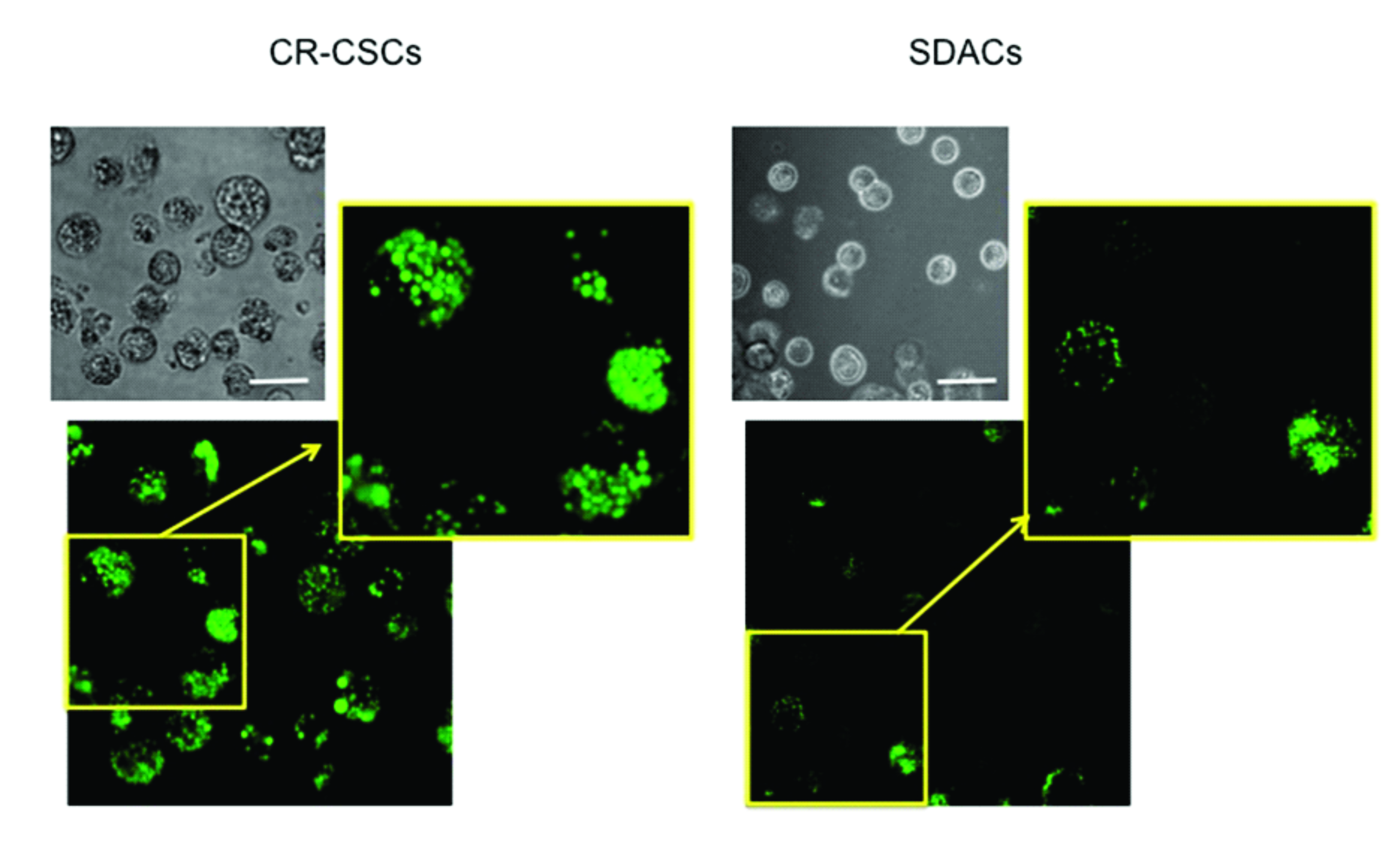

Supplement: Supplementary file 4 — Supporting Figure 4 [file stem0033-0035-sd4.tif]

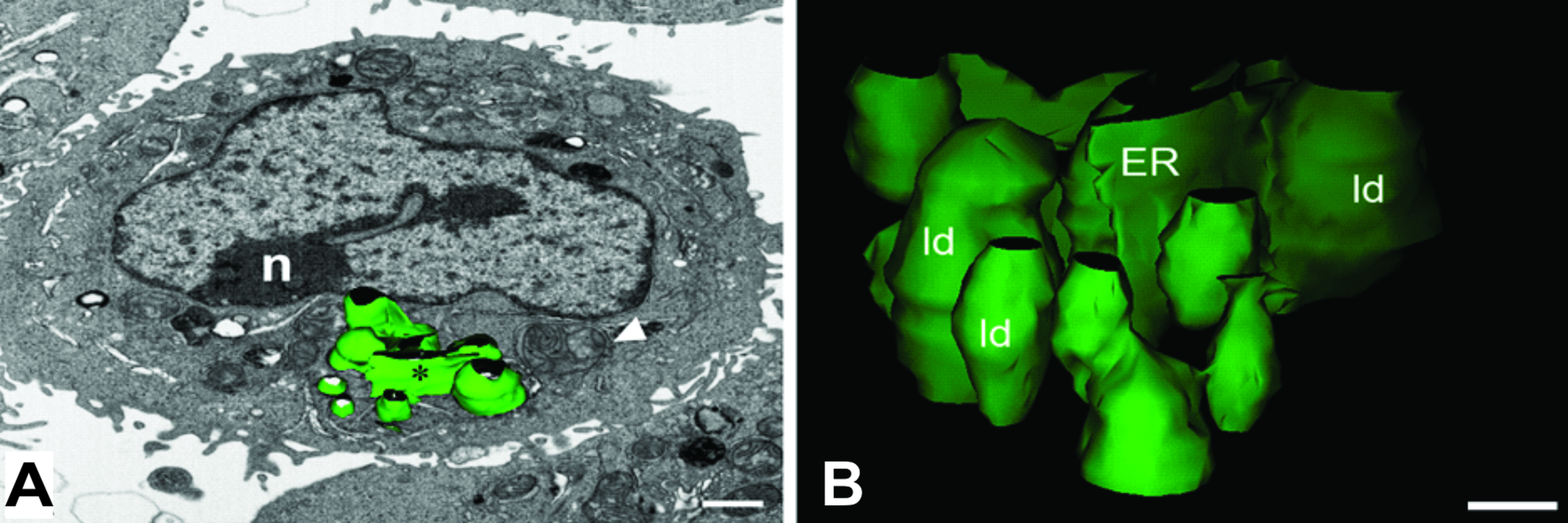

Supplement: Supplementary file 5 — Supporting Figure 5 [file stem0033-0035-sd5.tif]
