# Supplementary material for: Lipid Droplets: A New Player in Colorectal Cancer Stem Cells Unveiled by Spectroscopic Imaging
Source: Stem Cells. 2014 Dec 18;33(1):35–44. doi: 10.1002/stem.1837 (PMC4311668; doi:10.1002/stem.1837)
Supplement: Supplementary file 6 — Supporting Figure Legends [file stem0033-0035-sd6.doc]

**Supplementary Figure Legends**

**Fig. S1. CR-CSCs Raman detection through 830 nm laser power**

Cell-averaged Raman fingerprint for each colon cell line, performed with 830 nm laser source. CR-CSCs show a unique behavior at 1300 cm^-1^; the intensity of this Raman peak is clearly more pronounced than in other non-stem colon cancer cell lines.

**Fig. S2. Raman measurements on CR-CSCs from 6 different patients**

The average measurements (red curves) of CR-CSCs from six different patients are reported along with the standard deviations (gray shadows). The spectral acquisition is performed in the 800-1800 cm^-1^ range. All the curves exhibit a similar behavior with a pronounced intensity of the 1300 cm^-1^ Raman peak, thus confirming the reproducibility of this spectral feature.

**Fig. S3. Principal Component Analysis (PCA) performed on all colon cancer cells.**

Principal component analysis has been performed on the whole panel of cells at once, i.e. over all the cells simultaneously. The spectral data have been divided in the usual two regions, from 800 up to 1800 cm^-1^ and from 2800 up to 3100 cm^-1^. (**A**) The principal component PC2 results to be the one accounting for the differences occurring inside the cells. More in details, PC2 in the range 2800-3100 cm^-1^ exhibits a clear peak at 2850 cm^-1^ and a dip at 2950 cm^-1^, corresponding respectively to lipid and protein vibrations. For this reason, it is a good parameter for the lipid/protein ratio. Similarly the PC2 in the 800-1800 cm^-1^ shows positive lipid peaks at 1300, 1440, 1740 cm^-1^. (**B**) Besides the bright field images of the cells, we show the PC2 scores map for the mentioned spectral ranges: 400-1800 cm^-1^ on the left and 2800-3100 cm^-^1 on the right. The red areas are lipid rich ones, while the blue are proteins rich regions.

**Fig. S4. LDs detection through confocal-fluorescence microscopy**

Comparison of typical large field-of-view z-projected confocal fluorescence images of the investigated cell lines stained with BODIPY 493/503. CR-CSCs (left panels) over-express LDs, compared to the SDACs (right panels). Scale bar is 25 μm.

**Fig. S5. Endoplasmic reticulum-lipid droplets cluster as obtained by serial sectioning Focused Ion Beam (FIB) Scanning Electron Microscopy (SEM).**

(**A**) Side view of the surface rendered reconstructed volume superimposed over a roughly central cell slice, shown for perspective. n, nucleus. (**B**) Higher magnification of the surface rendered volume. One asterisk, LDs; two asterisks, endoplasmic reticulum. Scale bars in (**A**) and (**B**) are respectively of 1.2 and 0.5 µm.
