# Supplementary material for: Lipid Droplets: A New Player in Colorectal Cancer Stem Cells Unveiled by Spectroscopic Imaging
Source: Stem Cells. 2014 Dec 18;33(1):35–44. doi: 10.1002/stem.1837 (PMC4311668; doi:10.1002/stem.1837)
Supplement: Supplementary file 7 — Supporting Materials and Methods [file stem0033-0035-sd7.docx]

**Supplementary Materials and Methods**

**Cell Cultures**

Human colon carcinomas specimens were obtained from 7 patients (stage II-IV) undergoing colorectal resection, in accordance with the ethical standards of the institutional committee. Tumor diagnosis was based on anatomical and histopathological analysis. Tumor tissues were mechanically and enzymatically digested using collagenase (1.5 mg/mL; Gibco) and hyaluronidase (20 µg/mL; Sigma-Aldrich). The tumor digest was divided into different culture conditions to obtain different cell populations enriched in particular cell subsets. To obtain the 7 different CR-CSC samples, part of the tumor digest was cultured on ultralow adhesion flasks (Corning, Lowell, MA) in the presence of serum-free medium supplemented with epidermal growth factor (20 ng/mL) and basic fibroblast growth factor (10 ng/mL, both from Sigma-Aldrich, St. Louis, MO 63103, USA) to promote the growth of CSCs as spheres in 1–2 months, as previously described [1]. Tumor digest was also cultured in the presence of Dulbecco’s modified Eagle medium (EuroClone Ltd.) and supplemented with 10% heat-inactivated fetal bovine serum, Antibiotic/Antimycotic (Euroclone), Penicillin/Streptomycin (Euroclone), L-glutamine (Euroclone), to obtain primary tumor cells. Just the sphere cultures that were validated for a CR-CSCs phenotype and for the ability to form a xenograft in immune-compromised mice, resembling the parental tumor, were considered as CR-CSCs and were used for subsequent studies. To achieve the *in vitro* differentiation of CR-CSCs, dissociated sphere cells were cultured in Dulbecco’s modified Eagle medium supplemented with 10% FBS in adherent conditions for at least 25 days, obtaining four Sphere Derived Adherent Cells (SDACs) (from patients 1-4). Human CCD841-CoN normal epithelial colon cell line (ATCC CRL-1790) was cultured in α-Mem (Invitrogen) with 10% FBS (Invitrogen) and antibiotics (100 U/ml penicillin, 100 μg/ml streptomycin) (Invitrogen). Human HCT116 and RKO colorectal carcinoma cell lines (ATCC CCL-247 and ATCC CRL-2577 respectively) were cultured in RPMI (Invitrogen) with 10% FBS (Invitrogen) and antibiotics (100 U/ml penicillin, 100 μg/ml streptomycin) (Invitrogen).

**Raman spectra analysis**

Most of the Raman spectra presented in this work are average curves coming from a large number of measurements. In the case of spectra of Fig. 1B, the curves are computed averaging the spectra collected on the pixels of the map corresponding to the area of the cell. Instead Fig. S2 shows the results of a Raman study on 6 different CR-CSC samples, from 6 different patients. In this experiment single point Raman measurements are performed on single cells, with a larger laser spot-size so that nearly all the cell is probed at once. For each patient, at least 50 spectra are collected on different cells. Subsequently average spectra and corresponding standard deviations are calculated for each cell line.

Principal component analysis (PCA) is performed separately in the 800-1800 cm^-1^ and 2800-3100 cm^-1^ spectral regions (Fig. S3). In order to compare the results of this multivariate technique between the different cell lines (i.e.: NECCs, CR-CSCs, SDACs, CCCs), the recorded spectra are processed altogether as one single group at once, and consequently the computed principal components are exactly the same for all the cell lines. Pseudo-color maps are produced for a fast visualization of multivariate results by mapping the so-called scores of principal components as a function of the *xy* spatial coordinates.

**Raman peaks assignment**

The assignment of all the Raman shifts reported in the main text has been thoroughly discussed in the literature. The peaks at 1300 and 1740 cm^-1^ are unambiguously assigned to lipids molecular vibrations and correspond respectively to the CH_2_ twist and to the C=O in –CH_2_-COOR, this last due to triacylglycerol [2, 3]. The Raman band at 1440-1450 cm^-1^ is assigned to the CH_2_ bending, occurring at 1440 cm^-1^ for lipids and 1450 cm^-1^ for proteins [4]. Depending on the lipid to protein ratio, this peak shifts from 1440 to 1450 cm^-1^. Concerning the spectral region between 2800 and 3000 cm^-1^, CH_2_ symmetric and asymmetric stretching are respectively found at 2850 and 2885 cm^-1^, while the analogous vibrations for CH_3_ are located at 2933 and 2950 cm^-1^ [4]. Similarly to 1440-1450 cm-1 band, CH_2_ stretching intensities are suitable indicators for the lipid to protein ratio, due to the fact that CH_2_ groups are more frequent in fatty acids chains than in proteins. Finally, the band at 1050-1100 cm^-1^ correspond to C-C stretching vibrations in hydrocarbon chain [3].

**Raman spectroscopy as sorting tool for CR-CSCs**

The unambiguously difference of intensity of the 1300 cm^-1^ Raman peak offers the possibility to discriminate between CR-CSCs and the other cell types involved in the cancer progression. An 830 nm diode laser is used to perform Raman measurements over all the cell samples. Here the laser focus has not a point-like shape but an elliptical shape with one of the two axes much larger than the other. This kind of line-focus allows for probing the cell almost in its entirety with a single Raman measurement, thus saving a lot of time compared to Raman mappings. This is a crucial point for future applications in cancer stem cell sorting.

First, we measured Normal Epithelial Colon Cells (NECCs), Colorectal Cancer Stem Cells (CR-CSCs), Sphere Derived Adherent Cells (SDACs), and Colon Cancer Cells (CCCs) in the spectral region between 800 and 1800 cm^-1^. The differences highlighted in the main text for this spectral region were still evident in these measurements (Fig. S1), with the 1300 cm^-1^ peak reporting the most evident difference between the CR-CSCs on one side and the other cells types on the other side. Subsequently we have performed similar measurements on other six CR-CSCs lines from six different patients. For each patient, at least 30 cells are probed and Fig. S2 reports the average spectra with the standard deviations. For all the patients the peak at 1300 cm^-1^ is clearly more pronounced than the two side peaks at 1260 and 1340 cm^-1^ respectively, while in the other cell types of the cancer progression the intensity level of all the three peaks (1260, 1300 and 1340 cm^-1^) is comparable.

**Sample preparation for Electron Microscopy measurements**

NECCs, CR-CSCs, SDACs, and CCCs were processed as follows. Briefly the cell monolayers were fixed for 45 min at room temperature in 2% glutaraldehyde in 0.1 M cacodylate buffer (pH=7.4). The cells were then harvested from the Petri substrate with a cell scraper, transferred in Eppendorf tubes and centrifuged at 10000 g for 10 min. Once fixed in fresh fixative for 1 h at room temperature, the cell pellets were washed three times for 10 min in 0.1 M cacodylate buffer. Secondary fixation was carried out in 1% aqueous osmium tetroxide for 2 h at room temperature, followed by three washes, 10 min each, in 0.1 M cacodylate buffer. After two short washes in bi-distilled water, the specimens were stained overnight in 1% uranyl acetate in 70% ethanol at 4 °C, and then dehydrated through a graded series of ethanol. The cell pellets were then placed in propylene oxide for 15 min twice. Infiltration was accomplished by placing the pellets in a 1:1 mixture of propylene oxide and Epon resin for 4 h at room temperature. The pellets were then left in Epon resin for 2 h at room temperature, and finally embedded in fresh resin for 48 h at 65 °C.

**Transmission electron microscopy and stereological analysis**

Sections of about 70 nm were cut with a LeicaEMUC7 ultramicrotome, stained with 1% uranyl acetate in bi-distilled water and lead citrate and observed with a JEOL JEM 1011 electron microscope operating at an accelerating voltage of 100 KV. Images were recorded with an 11 Mp GatanOrius SC100 Charge-Coupled Device (CCD) camera.

The volume fraction of the cell occupied by lipid droplets was estimated using point counting stereology techniques [5]. For each cell sample, 5 arbitrarily selected sections were systematically random sampled and quantified at a magnification of 4,000 to unambiguously recognize LDs. Sections and areas were chosen such that no cell was sampled more than once. The number of cells in any given area varied, but the total cellular area sampled averaged 2,700 mm^2^ for each sample. The mean and standard error were calculated for each grid (n = 5). Comparison of the volume fraction of cell occupied by LDs for each sample was done with Student’s t-test.

**Focused ion beam milling and the acquisition of serial scanning electron microscopy images**

The block preparation for serial section scanning electron microscopy was performed following Knott et al. [6, 7]. The block was observed in a Dual-Beam microscope (FEI Helios NanoLab), The sections, with an average thickness of around 100 nm, were milled using an acceleration voltage of 30 keV and a current of 1000 pA. After the removal of each layer, images were collected from a smaller subregion at a magnification of 13 nm/pixel, and with a total image size of 1024 x 943 pixels (943 KB/image). This gave a horizontal field width of 16.5 µm. The acceleration voltage of the imaging beam was 2 keV, with a current of 200 pA and dwell time of 30 μs/pixel.

**SI References**

1. Ricci-Vitiani, L., et al., *Identification and expansion of human colon-cancer-initiating cells.* Nature, 2007. **445**(7123): p. 111-115.

2. Wu, H., et al., *In vivo lipidomics using single-cell Raman spectroscopy.* Proceedings of the National Academy of Sciences of the United States of America, 2011. **108**(9): p. 3809-3814.

3. Chan, J.W., et al., *Raman spectroscopic analysis of biochemical changes in individual triglyceride-rich lipoproteins in the pre- and postprandial state.* Analytical Chemistry, 2005. **77**(18): p. 5870-5876.

4. Krafft, C., et al., *Identification of organelles and vesicles in single cells by Raman microspectroscopic mapping.* Vibrational Spectroscopy, 2005. **38**(1-2): p. 85-93.

5. Howard, C.V.R., M.G. , *Unbiased Stereology. Three-Dimensional Measurement in Microscopy.* Oxford: BIOS Scientific Publishers, 1998.

6. Knott, G., et al., *Serial Section Scanning Electron Microscopy of Adult Brain Tissue Using Focused Ion Beam Milling.* The Journal of Neuroscience, 2008. **28**(12): p. 2959-2964.

7. Knott, G., et al., *Focussed Ion Beam Milling and Scanning Electron Microscopy of Brain Tissue.* J Vis Exp, 2011(53): p. e2588.
